# Is it Worth Adding Systemic Antibiotics to Inhalational Tobramycin Therapy to Treat Pseudomonas Infections in Cystic Fibrosis?

**DOI:** 10.7759/cureus.17326

**Published:** 2021-08-20

**Authors:** Kumar Karki, Santosh Sigdel, Sunam Kafle

**Affiliations:** 1 Internal Medicine, Larkin Community Hospital, Miami, USA; 2 Internal Medicine, Avera McKennan Hospital and University Health Center, Sioux Falls, USA; 3 Internal Medicine, College of Medical Sciences, Bharatpur, NPL

**Keywords:** ‘cystic fibrosis’, ‘inhaled tobramycin’, ‘pseudomonas aeruginosa’, ‘pulmonary infection’, ‘systemic antibiotics’, and ‘oral antibiotics’

## Abstract

*Pseudomonas aeruginosa* (PA), a gram-negative rod-shaped bacterium, is one of the most common pathogens causing colonization and infection of the respiratory tract and lungs in patients with cystic fibrosis (CF). Antibiotic therapy is the mainstay treatment for PA infection, and tobramycin is one of the widely used antibiotics in intravenous or inhalation form. This review aims to explore if there is any advantage of adding systemic antibiotics to tobramycin inhalation therapy by comparing the combination regimen to tobramycin inhalation monotherapy in CF patients with PA infection. We collected studies relevant to our review topic by doing a literature search on multiple databases. According to the currently available studies, the addition of oral antibiotics such as fluoroquinolones and azithromycin to tobramycin inhalation solution (TIS) provides no additional benefit in eradicating PA infection or producing clinical improvement in cystic fibrosis patients. However, adding intravenous antibiotics to TIS has not produced conclusive results and thus requires further research. We recommend conducting more randomized controlled trials comparing different treatment regimens, which may help discover the most beneficial treatment regimen with decreased systemic side effects.

## Introduction and background

Cystic fibrosis (CF), a common disease among people of northern European descent, is an autosomal recessive genetic disorder [1]. The incidence of CF is estimated between 1 in 3,000 to 1 in 6,000 live births [2,3]. The cause of this disorder is a mutation in the cystic fibrosis transmembrane regulator (CFTR) protein-coding gene [1]. CFTR helps transmembrane chloride ions transport through cyclic AMP (cAMP)-activated chloride channels [1]. The abnormal chloride secretion in CF affects multiple organs, notably the upper and lower respiratory tracts, pancreas, bowel, reproductive tract, and causes sinopulmonary infections, pancreatic insufficiency, infertility [1,4]. Among these complications, pulmonary infections are the most common cause of morbidity and mortality in CF patients [1,4].

Defect in CFTR cause abnormal secretion of chloride and bicarbonate ions in the airway resulting in hyper-concentrated mucus, mucus adhesion to the respiratory surface, and defective mucus transport [5,6]. This leads to impaired mucociliary clearance and an increased risk of bacterial colonization and infection in the respiratory tract, including the lungs [6]. *Pseudomonas aeruginosa* (PA) is a common pathogen causing lung infections in patients with CF [7]. A specific pattern of lung infections occurs from early childhood in these patients [7,8]. Pulmonary infections with Staphylococcus aureus usually occur in the early years of life, followed by respiratory colonization with *P. aeruginosa* later in childhood [7,8]. Chronic endobronchial colonization by *P. aeruginosa* causes slowly increasing tissue damage, ultimately leading to accelerated disease progression, making it one of the clinically significant respiratory pathogens in CF patients [7,8].

Treatment with oral, intravenous (IV), or inhaled formulations of various antibiotics are currently used to manage Pseudomonas infections in CF patients [9]. The inhalation route can deliver relatively high doses of antibiotics directly to the site of infection with minimum systemic exposure, low risk of toxicity, and can achieve sufficient local concentrations of the drug to kill the pathogen [10]. Tobramycin inhalation solution (TIS) is given in a nebulized aerosol form to treat pulmonary infections in CF patients [10]. A randomized controlled trial (RCT) performed by Mogayzel Jr et al. and the ELITE trial are some of the studies demonstrating the efficacy of TIS for treating early PA infection in patients with CF [11,12]. According to the cystic fibrosis foundation (CFF), inhaled antibiotics, preferably inhaled tobramycin, are the preferred treatment modality for initial Pseudomonas infection in CF [9]. CF patients have frequent pulmonary exacerbations treated with oral, IV, or inhaled antibiotics [13]. In this review, we aim to ascertain if the addition to TIS therapy of an oral or IV antibiotic against *P. aeruginosa* proves to enhance the efficacy of TIS against *P. aeruginosa* infection in CF patients.

## Review

Methods and result

We searched for related articles on multiple databases such as PubMed, Scopus, Embase and found articles relevant to our review. We used the keywords 'cystic fibrosis', 'inhaled tobramycin,' 'pseudomonas aeruginosa,' 'pulmonary infection,' 'systemic antibiotics,' and 'oral antibiotics' in various combinations with Boolean operators 'AND' and 'OR' to do the literature search. A total of eight articles relevant to our review question were included in this literature review. We have included articles comparing the TIS monotherapy to TIS plus systemic antibiotics dual therapy. There were six RCTs and two retrospective studies relevant to this review topic. The characteristics of the included studies are mentioned in Table 1. 

**Table 1:** Characteristics of the included studies. TIS: tobramycin inhalation solution; PA: *Pseudomonas aeruginosa*; IV: intravenous; BALF: bronchoalveolar lavage; FQ: fluoroquinolone; CF: cystic fibrosis; PO: per oral; mg: milligrams; RCT: randomized controlled trial.

SN	Study	year	Study design	Participants	Age group	Interventions	Comparator	Outcome
1	Jablonski et al. [14]	2020	Retrospective cohort study	75 CF patients with positive PA culture	<21 years	TIS 300mg twice daily	1. TIS plus FQ, 2. TIS plus IV tobramycin	There was no significant difference between PA eradication rate and recurrence rate in patients receiving TIS compared to TIS+FQ. Time to first follow-up culture was significantly shorter for patients who received TIS+IV antipseudomonal therapy compared to other regimens
2	Choi et al. [15]	2020	Retrospective study	18 CF patients with initial infection with PA	<1 year	TIS	TIS plus oral ciprofloxacin	There was no significant difference in the rates of PA eradication between the two regimen
3	Mayer-Hamblett et al. [16]	2018	RCT	221 children with CF with new PA infection	6 months-18 years	TIS 300mg BD + Azithromycin PO 10mg/kg thrice a week	TIS 300mg twice daily + placebo	There was a decreased risk of pulmonary exacerbation in children receiving azithromycin compared to placebo
4	Nichols et al. [17]	2016	RCT	176 CF patients with chronic PA. (128/176 were under azithromycin treatment)	NA	4 weeks of TIS followed by 4 weeks of inhaled aztreonam	4 weeks of TIS followed by 4 weeks of placebo	Azithromycin is associated with a reduction in the ability of inhaled tobramycin to improve quality of life and lung function, as compared to inhaled aztreonam
5	Nick et al. [18]	2014	Clinical trial	263 CF patients with reported baseline PA in the sputum sample	NA	inhaled tobramycin	Inhaled tobramycin plus oral azithromycin	Patients receiving TIS and azithromycin showed the earlier need for additional antibiotics, decreased disease-related quality of life improvement, and a less likely reduction in sputum P. aeruginosa density than those receiving only TIS
6	Treggiari et al. [19]	2011	RCT	304 children with CF and sputum culture positive for PA	1-12 years	TIS (300 mg twice a day) for 28 days plus oral ciprofloxacin (15-20 mg/kg twice a day)for 14 days	TIS (300 mg twice a day) for 28 days plus oral placebo for 14 days	There was no statistical difference between pulmonary exacerbations between ciprofloxacin and placebo groups. There were more side effects, such as increased cough in the group receiving ciprofloxacin
7	Noah et al. [20]	2010	Randomized, prospective study	21 children with CF and positive surveillance culture for PA	6 months -16 years	TIS 300 mg twice daily for four weeks	IV ceftazidime and TIS 300mg for two weeks	Both treatment regimens appeared to reduce the concentration of PA in BALF, and there was no significant difference between the two treatment regimens

Discussion 

Tobramycin, colistin, and aztreonam are the currently available inhalational antibiotics to treat chronic or newly acquired PA infection in patients with cystic fibrosis [21]. When comparing the nebulization forms of tobramycin and colistin, tobramycin has shown to be more effective in improving lung function in these patients [22]. However, these two antibiotics had equivalent results in reducing the bacterial load and safety profile [22]. Tobramycin can be given as tobramycin inhalation solution, tobramycin inhalation powder (TIP), or nebulized concentrated tobramycin [21]. The inhaled form of tobramycin has demonstrated improvement in lung function, reduced the concentration of P. aeruginosa in sputum, and decreased the risk of future hospitalizations in CF patients [23]. It is not associated with the usual side effects of aminoglycosides, such as renal and audiological complications [23]. Tobramycin, given in inhalation form to maximize efficacy while minimizing the toxic effects, is one of the most effective antibiotic regimens currently available to treat PA infection in CF. 

Comparison Between TIS and TIS Plus Fluoroquinolone

Inhaled tobramycin as a treatment option for CF patients with initial positive culture for PA was compared to the combination of TIS and oral fluoroquinolone in three studies [14,15,19]. Treggiari et al. conducted an RCT including 304 children aged one to twelve years, comparing the efficacy of TIS plus ciprofloxacin to TIS plus placebo [19]. This study showed no statistically significant difference between these two regimens' pulmonary exacerbation (PE) rates [19]. However, cough was reported more frequently in the group receiving ciprofloxacin [19]. Likewise, two retrospective studies compared the effects of TIS monotherapy with TIS plus fluoroquinolone dual therapy [14,15]. The study by Choi et al., which included only 18 infants, demonstrated no significant statistical difference in the PA eradication rates between the infants receiving TIS monotherapy compared to those receiving TIS plus ciprofloxacin dual therapy [15]. The time for re-infection with PA was also similar between the two treatment groups [14]. The study performed by Jablonski et al. also had similar results showing no statistical difference in the eradication rates of PA infection between the two groups [14]. 

In retrospective studies, the results of the interventions cannot be accessed over time unlike in prospective studies, so the comparison of outcomes of the above-discussed retrospective studies with other available studies in literature should be made with caution [14,15]. Also, both these studies had a small sample size for the patients receiving systemic antibiotics, and they were single-center studies, so that the findings may have been impacted by selection bias [14,15]. However, the study by Treggiari et al. was a large multicenter RCT, and its outcome was similar to the above retrospective studies [14,15,19]. Therefore, we suggest clinicians reconsider adding FQ antibiotics such as ciprofloxacin as an add-on therapy to TIS since it does not seem to provide any added clinical benefit.

Comparison Between TIS and TIS Plus Azithromycin

Mayer-Hamblett et al. conducted an RCT comparing TIS and azithromycin therapy to TIS plus placebo [16]. This study included 221 children aged six months to 18 years suffering from CF who had newly acquired PA infection [16]. The number of children who developed pulmonary exacerbations (PE) was 43/110 in the azithromycin group and 58/111 in the placebo group [16]. There was a 44% overall decreased risk of PE in children receiving azithromycin plus TIS compared to TIS alone, which was statistically significant [16]. In contrast, the study by Nick et al. showed that the group of patients receiving azithromycin plus TIS had decreased improvement in disease-related quality of life and an earlier need for additional antibiotics than the group receiving TIS therapy alone [18]. Likewise, Nichols et al. conducted a study comparing inhaled tobramycin plus inhaled aztreonam to inhaled tobramycin plus placebo [17]. Nearly two-thirds of the study participants were under azithromycin treatment throughout the whole study duration [17,18]. In the patients receiving azithromycin, the lung function improved significantly less in the first four weeks of receiving TIS compared to the subsequent four weeks of receiving aztreonam [17]. They concluded that using azithromycin reduced the ability of inhaled tobramycin compared to inhaled aztreonam to improve the patients' quality of life and lung function [17]. Recent literature from the CFF has reported the results of the TEACH trial [24]. In this trial, patients with CF who had chronic airway infection with PA were treated with TIS in addition to either azithromycin or placebo, and there was no difference in the rates of acute PE, need for hospital admission, or additional antibiotic requirement between the participants receiving azithromycin and placebo [24]. 

Azithromycin has an anti-inflammatory effect on various pulmonary diseases, including diffuse panbronchiolitis, asthma, bronchiectasis, cystic fibrosis [25]. Two different clinical trials found azithromycin to decrease PE requiring antibiotics and improve or stabilize pulmonary function in patients with CF [26,27]. However, the efficacy of azithromycin as an add-on antibiotic to inhaled tobramycin therapy in treating PA infection in CF patients remains controversial. Studies have demonstrated different outcomes, including increased clinical benefit, no added benefit, or decreased clinical benefit from adding azithromycin to TIS therapy. We, therefore, suggest larger scale RCTs with longer study duration be conducted to reach a better and conclusive result.

Comparison Between TIS and TIS Plus IV Antibiotics 

Antibiotics such as ceftazidime or piperacillin plus aminoglycoside combination have been the preferred treatment choice for severe PA infections, especially in intensive care units [28]. However, for newly acquired and chronic PA infections, inhaled forms of antibiotics such as tobramycin are the preferred modalities of treatment [28]. A study by Jablonski et al. compared the efficacy of TIS therapy to TIS plus IV tobramycin in CF patients with initial PA infection [14]. The results showed that the recurrence rates and time to recurrence were not significantly different between the two groups; however, all patients receiving IV antibiotics in combination with TIS achieved eradication, unlike those receiving TIS monotherapy [14]. Noah et al. also conducted a study comparing the efficacy of TIS monotherapy with TIS plus IV ceftazidime therapy [20]. The study found that both treatment modalities had similar effects on reducing bacterial burden as demonstrated by PA concentration in BALF, but systemic antibiotics had a more significant impact in decreasing lower airway inflammation [20].

This review analyzed different studies that compared TIS to the combination of TIS and oral or IV antibiotics used in initial or chronic PA infection in CF. Since most of the studies showed minimal or no difference in the efficacy between these two treatment regimens, we recommend TIS inhalation monotherapy to treat PA infections in CF patients without the addition of oral or IV antibiotics. Our review has some limitations, though. We extracted data from only seven studies. Also, the age group and the inclusion criteria for the study population and the measured outcomes were not the same in the discussed studies. Some studies had included patients with newly acquired PA infection, and some had patients with chronic PA infection as their study population. In addition, there was no uniformity in the doses and duration of treatment. Thus, the findings of these studies should be interpreted with careful consideration. There was only one study that included the adult population. Large-scale studies with patient populations of different age groups should be conducted, which may help develop better patient-centric guidelines based on better scientific evidence.

## Conclusions

We discussed the efficacy of inhaled tobramycin compared to combined tobramycin inhalation and systemic antibiotics to treat initial or chronic Pseudomonas infections in cystic fibrosis patients. Current literature suggests minimal or no added benefit of adding intravenous antibiotics and no additional benefit of adding oral antibiotics to tobramycin inhalation therapy. Moreover, the addition of systemic antibiotics poses the risk of undesirable side effects not seen in the TIS monotherapy regimen. Therefore, we recommend inhalation therapy with tobramycin be used as monotherapy for initial or chronic PA infection in CF patients.
